# The cross-cutting contribution of the end of neglected tropical diseases to the sustainable development goals

**DOI:** 10.1186/s40249-017-0288-0

**Published:** 2017-04-04

**Authors:** Mathieu Bangert, David H. Molyneux, Steve W. Lindsay, Christopher Fitzpatrick, Dirk Engels

**Affiliations:** 1 Department of Control of Neglected Tropical Diseases, World Health Organization, 20 Avenue Appia, 1211 Geneva, Switzerland; 2 Department of Parasitology, Liverpool School of Tropical Medicine, Liverpool, UK; 3 Department of Biosciences, Durham University, Durham, UK

**Keywords:** Neglected tropical diseases, Sustainable development goals

## Abstract

The Sustainable Development Goals (SDGs) call for an integrated response, the kind that has defined Neglected Tropical Diseases (NTDs) efforts in the past decade.

NTD interventions have the greatest relevance for SDG3, the health goal, where the focus on equity, and its commitment to reaching people in need of health services, wherever they may live and whatever their circumstances, is fundamentally aligned with the target of Universal Health Coverage. NTD interventions, however, also affect and are affected by many of the other development areas covered under the 2030 Agenda. Strategies such as mass drug administration or the programmatic integration of NTD and WASH activities (SDG6) are driven by effective global partnerships (SDG17). Intervention against the NTDs can also have an impact on poverty (SDG1) and hunger (SDG2), can improve education (SDG4), work and economic growth (SDG8), thereby reducing inequalities (SDG10). The community-led distribution of donated medicines to more than 1 billion people reinforces women’s empowerment (SDG5), logistics infrastructure (SDG9) and non-discrimination against disability (SDG16). Interventions to curb mosquito-borne NTDs contribute to the goals of urban sustainability (SDG11) and resilience to climate change (SDG13), while the safe use of insecticides supports the goal of sustainable ecosystems (SDG15). Although indirectly, interventions to control water- and animal-related NTDs can facilitate the goals of small-scale fishing (SDG14) and sustainable hydroelectricity and biofuels (SDG7).

NTDs proliferate in less developed areas in countries across the income spectrum, areas where large numbers of people have little or no access to adequate health care, clean water, sanitation, housing, education, transport and information. This scoping review assesses how in this context, ending the epidemic of the NTDs can impact and improve our prospects of attaining the SDGs.

**Electronic supplementary material:**

The online version of this article (doi:10.1186/s40249-017-0288-0) contains supplementary material, which is available to authorized users.

## Multilingual abstracts

Please see Additional file 1 for translations of the five official working languages of the United Nations.

## Background

The 17 goals of the SDG agenda integrate all three dimensions of sustainable development: the economic, social and environmental, around the themes of people, planet, prosperity, peace and partnership. Following on from the Millennium Development Goals (MDGs), the Sustainable Development Goals (SDGs) prioritize the fight against poverty and hunger, while also focusing on human rights for all, and the empowerment of women and girls as part of the momentum to achieve gender equality. They also build upon the MDGs in order to address the “unfinished business” of the MDG timeframe. The SDGs recognize that eliminating poverty and inequality, creating inclusive economic growth and preserving the planet are inextricably linked, not only to each other, but also to the health of populations. They also acknowledge the dynamic and reciprocal relationships between each of these elements. For example, a fundamental assumption of the SDGs is that health is a major contributor and beneficiary of sustainable development policies [1].

The Neglected Tropical Diseases (NTDs) are a diverse group of 17 human and zoonotic diseases whose health and economic burden falls most heavily on the poorest people and communities [2] NTDs include diseases such as soil-transmitted helminths (STH), lymphatic filariasis, onchocerciasis and schistosomiasis, as well as dengue and rabies to list a few. Within the SDGs, the NTDs have been included as a target to “end the epidemic of NTDs by 2030” [3], an important achievement since the inception of this grouping of diseases [4, 5], which have been recently described as a “chronic pandemic” affecting many millions [6]. Requirement for mass and individual treatment or care for NTDs, a proxy indicator of NTD prevalence, offers important insights into the way the NTD burden is shared. In 2014, at least 1.7 billion people required mass or individual treatment and care for NTDs in 185 countries. Of these, 1.1 billion were in lower- or middle-income countries. This is not to minimise the burden in the least developed countries. All low-income countries are affected by at least five NTDs [2], while the 520 million people requiring treatment in low-income countries represent a staggering 60% of those countries’ populations.

The NTDs and responses to them have the most direct relevance for the health goal, but they also have wide, cross-cutting and cross-sectoral linkages and effects that make them relevant to all the SDGs [7]. Thus efforts to mitigate their impact will have a direct influence on overall SDG progress [8].

## Main text

### SDG 1. End poverty in all its forms everywhere

SDG 1 is among the most ambitious of the 17 goals. According to the 2030 Agenda, ending poverty in all forms and dimensions by 2030 involves targeting those living in vulnerable situations, increasing access to basic resources and services, and supporting communities affected by conflict and climate-related disasters. Rates of extreme poverty have declined by more than half since 1990, in part, as a result of the rebalancing of global trade towards Asia. However, one in five people in developing regions still live on less than $1.25 a day, and there are millions more who survive on little more than this daily amount. The 2030 Agenda underlines the fact that poverty is more than the lack of income and resources to ensure a sustainable livelihood.

The disabling and debilitating effects of NTDs prevents adults from providing for their families and contributing to the economic development of their countries, and generates a significant care burden. At the household level, this results in generations becoming trapped in a cycle of increased medical costs, poverty and disease. The so-called medical poverty trap affects people worldwide, examples including countries such as Cambodia and Viet Nam, where 50–67% of affected households have incurred debt as a result of treatment for dengue [9], or in Bangladesh [10] and Nepal [11], where 25–75% of households affected by visceral leishmaniasis experience some type of financial catastrophe in obtaining a diagnosis and treatment. NTDs are a financial burden on patients even when tests and medicines are provided free of charge [12, 13], highlighting the impact these diseases have beyond clinical care. The impact of NTDs on the workforce can be enormous. The chikungunya epidemic on Reunion Island resulted in 112 400 lost days of work from 12 800 cases, costing an estimated $18 million [14]. Families also pay a heavy price in the form of out-of-pocket costs incurred at the time of seeking care for treatment. In Ghana, the cost of care per patient with Buruli ulcer in a household in the poorest earning quartile can be as high as 315% of annual earnings [15]. Similarly, in Nigeria the cost of Buruli Ulcer care and diagnosis can be on average 162% of patient’s median monthly household income, leading to catastrophic financial loss [16]. The cost of NTDs is not limited to human infections. Pigs are frequently kept as a cash reserve to be used in an emergency such as for medical needs. A study in Tanzania measuring the impact of cysticercosis infections on these “cash reserves” found a significant economic burden resulting from the reduced value or condemnation of pigs harbouring cycticercosis [17].

It is important to note that NTDs are diseases of poverty rather than diseases of poor countries. Their impact is felt by poor communities everywhere, including those living in wealthy countries. Indeed, many of the highest disease burden NTDs (just five NTDs represent 71% of the total NTD burden [18]) occur predominantly in the largest emerging market economies that comprise the group of 20 nations (G20), in addition to Nigeria, which also has one of the world’s largest economies [19]. Brazil, China, India, Indonesia, and Nigeria have the largest NTD prevalence, but even very wealthy nations, such as the United States of America (USA), have a hidden burden of NTDs, mostly concentrated in the southern states [20] as well as Australia, where blinding trachoma and scabies remain a major public health problems in Aboriginal communities [21]. In Europe, the Mediterranean basin is endemic for leishmaniasis [22]. Autochthonous outbreaks of dengue have also been reported in high-income countries of Portugal [23], France [24], Australia, USA, Singapore and Chinese Taipei [25] whilst recently a focus of urogenital schistosomiasis transmission (due to *Schistosoma haematobium*) was found in Corsica, France [26].

NTD programmes play an important role in reducing the financial burden on families seeking care both in the way interventions are delivered (i.e., free of charge and often through community directed interventions) and by placing greater emphasis on preventive care. Simply by preventing the development of disease, NTD programmes reduce exposure to the debilitating physical and mental health effects of NTDs [27] which give rise to catastrophic and impoverishing costs [28]. It seems clear, therefore, that progress on alleviating the burden of NTDs is a prerequisite for progress towards SDG 1.

### SDG 2. End hunger, achieve food security and improved nutrition and promote sustainable agriculture

SDG 2 aims to end all forms of hunger and malnutrition by 2030, ensuring that everyone, but especially children and the more vulnerable, have access to sufficient, nutritious food year round thereby ensuring food security. This will require promoting sustainable agricultural practices, improving the livelihoods and capacities of small-scale farmers, allowing equal access to land, technology and markets. It will also require international cooperation to ensure investment in infrastructure and technology to improve agricultural productivity.

NTDs have an impact on nutrition both directly and indirectly. Direct impacts include parasites such as soil-transmitted helminthiases (STH) consuming the nutrients required to keep people healthy [29]. These helminths compete for nutrients within the host and will naturally reduce the impact of food aid and other forms of nutritional transfers. The nutritional impairment caused by schistosome and STH infections during childhood has been shown to have an impact on the growth and development of children [30]. Anaemia and malnutrition are common side effects of several NTDs [31]. Food animals are also affected. Ruminants may fail to put on weight as a result of helminth infections [32], and cows produce up to 15% less milk as a result of nematode infections [33]. Trypanosomiasis in domestic animals, particularly in cattle, causes serious economic losses in livestock from anaemia, loss of condition and emaciation and is sometimes fatal resulting in loss of capital to poor livestock owners [34]. To a lesser extent, but nonetheless frequently fatal, outbreaks from orally-transmitted Chagas in fruit juices have affected food security in Venezuela [35].

Indirect impacts include the debilitating effects on those living with disease, such as farmers who are less able to work and thus produce the crops needed to feed themselves and their families, and impoverished consumers who are less able to pay for the food they need. It is fitting that Guinea worm disease translates to ‘the disease of the empty granary’ in Bambara, spoken by Dogon people in Mali, since the peak transmission period coincides with the harvesting season [36]. In addition, trichiasis and onchocerciasis prevents women from working, including fulfilling their traditional role as primary food providers due to visual impairment [37], making it difficult to work, socialise and even to walk beyond their homes [38].

Controlling and eliminating NTDs thus contributes to improving agricultural productivity, increasing food security and the nutritional status of affected communities. Cost-effective interventions of proven efficacy include the periodic deworming of children [39] together with improvement of water and sanitation [40], and health education [41], all of which have been shown to reduce the transmission of schistosome and soil-transmitted helminth infections [42–44]. The World Health Organization (WHO) recommends periodic treatment with anthelminthic (deworming) medicines, without previous individual diagnosis to all at-risk people living in endemic areas, including, preschool and school-aged children, and women of childbearing age, including pregnant women in the second and third trimesters and breastfeeding women. Treatment should be given once a year when the prevalence of STH infections in the community is over 20%, and twice a year when the prevalence of STH infections in the community exceeds 50%. The latest meta-analysis on de-worming and child nutrition confirms the consensus view regarding cost effectiveness of mass drug administration (MDA) [45].

With regard to agriculture, evidence of the beneficial impact of NTD interventions on nutrition include a study carried out in 2014 which shows that the 600 million people dependent on healthy livestock affected by zoonotic NTDs, such as cysticercosis, would derive multiple benefits by integrating sustainable management of helminth infections into the whole-farm economic context using a combination of helminth laboratory diagnostics and animal health economics. The study concluded that benefits would include increases in the safety and quality of food and an increased return on investment in food security [32].

### SDG 3 Ensure healthy lives and promote well-being for all

The health goal, the goal for which NTDs and NTD progress have the greatest relevance is discussed at length elsewhere [46]. Here, we limit ourselves to noting that the health goal has 13 targets, several of which are more or less direct transfers from the MDGs, but that the target for infectious diseases (3.3) has been expanded to include hepatitis, waterborne infections, and the NTDs. Passed over in the MDGs as “other diseases”, NTDs have now been accorded a specific target, reflecting their importance in terms of global prevalence and their social, economic and developmental consequences.

One important addition to the health goal of the SDGs is the inclusion of the Universal Health Coverage (UHC) target (3.8), the only target that cuts across all targets of the health goals, as well as addresses linkages with health-related targets in the other goals. Ensuring that essential services reach all those who need them is at the heart of NTD response efforts and is a fundamental component of WHO’s “Roadmap for implementation” of the 2012 NTD Strategy [47] endorsed by the World Assembly Resolution of 2013 [48]. Economists from 44 countries have called on global policy makers to prioritise a pro-poor pathway to universal health coverage (UHC) as an essential pillar of development [49]. NTD strategies, programmes and interventions are closely aligned with UHC goals or have components that have relevance for UHC. These include: defining and delivering an essential package of quality interventions across the full continuum of services; expanding coverage of services to ensure they reach all who need them; and providing financial protection to minimize out-of-pocket (OOP) payments and financial hardship. The notion of equitable access is woven into the fabric of the NTD agenda, serving as a constant reminder that cost-effectiveness is not the sole criterion for prioritisation of services, and that explicit consideration must be given to the most disadvantaged groups, including the low-income, rural and marginalised communities most at risk of NTDs.

### SDG 4 Ensure inclusive and equitable quality education and promote lifelong learning opportunities for all

Since 2000, there has been significant progress in achieving the target of universal primary education. The total enrolment rate in developing regions reached 91% in 2015, and the worldwide number of children out of school worldwide has dropped by almost half. There has also been a dramatic increase in literacy rates and many more girls are in school now than ever before. However, despite these advances, challenges remain, and, going forward, progress will depend on all girls and boys having access to free, quality primary and secondary education, and equal access to affordable vocational training. It will also require equal access to all levels of education and vocational training for the vulnerable, including people living with disabilities, indigenous peoples and children in vulnerable circumstances. The SDG focus on achieving quality education for all reaffirms the belief that education is one of the most powerful drivers of sustainable development.

That NTDs have a direct impact on school attendance and student performance is well established. For example, multiple studies have described the impact of STH and schistosome infections in children, reducing both school performance and attendance [50–52]. As many as two billion people are estimated to suffer from intestinal worms such as roundworm, hookworm, or whipworm [53], the largest burden of disease being found in sub-Saharan Africa and South Asia. STHs continue to have a major impact on public health, particularly in rural communities and particularly in regard to children’s school participation [54] and cognitive ability [55]. The indirect effects of NTDs on education, include the stigma and exclusion associated with disfiguring diseases [56].

Fortunately, a single dose of the inexpensive, safe and easily administered drugs albendazole and mebendazole is effective in the treatment of worms. The fact that the drugs have no significant side effects for uninfected children – and the fact that screening children for infection is much more expensive than treating them – has pointed policymakers towards a policy of mass treatment in high prevalence populations, including school age children [57]. School attendance increases for several reasons, some direct and related to morbidity. For example, deworming has been shown to improve cognition both in the short- and long-term (i.e., in communities 7–10 years after treatment) [58, 59].

School-based delivery of anthelminthics is one of the most cost-effective interventions to boost school attendance and performance as evidenced by a number of studies highlighting their positive impacts on children’s health, nutritional status, cognitive function and educational achievement [60]. A notable success story in this respect is a school-based deworming programme in Kenya which reduced absenteeism by 25%, and added a year to the average duration of a child’s education [51]. A long-term study revealed that the impacts of school-based deworming after 10 years included males staying enrolled for more years of primary school, working 17% more hours each week, spending more time in non-agricultural self-employment, being more likely to hold manufacturing jobs, and missing one less meal per week. The same study showed women being approximately one quarter more likely to have attended secondary school, thus halving the gender education gap [61].

The verdict from this body of evidence is clear: deworming treatment is not only highly effective and inexpensive, it is easy to administer through schools and brings benefits to children years after treatment [62]. There is a need to measure these benefits using robust epidemiological methods [63], while avoiding the biases such studies can entail [64, 65], in order to provide adequate evidence to policy makers. Nevertheless, with hundreds of millions of children still at risk of worm infection worldwide, providing free school-based deworming treatment is an easy policy “win” for health, education, and development.

### SDG 5. Achieve gender equality and empower all women and girls

Ending discrimination against women and girls is not only the fulfilment of a basic human right, but has implications for all SDG development areas. The last 15 years have seen some progress. For example, more girls are now in school compared to the year 2000, and most regions have reached gender parity in primary education. Women now make up to 41% of paid workers outside of agriculture, compared to 35% in 1990. The SDGs aim to build on these achievements to ensure that there is an end to discrimination against women and girls everywhere. The areas of focus are: access to paid employment; unequal treatment of women in the labour market; sexual violence and exploitation; harmful traditional practices such as child, early and forced marriage and female genital mutilation; ensuring universal access to sexual and reproductive health; affording women equal rights to economic resources such as land and property; and encouraging women leaders across all regions to strengthen policies and legislation for greater gender equality.

While NTDs impose a heavy burden on both sexes, there has been increasing recognition of the disproportionate impact of some NTDs on the health of girls and women [66]. Schistosomiasis is a matter of particular concern. Whether or not women are more exposed to schistosomiasis infection than men is unclear, evidence having been presented on both sides of the argument. On the one hand Huang and co-workers pointed out that the social and occupational roles may place men at an increased risk of acquiring certain NTDs, including schistosomiasis [67]. Whilst McDonald and co-workers found that gendered division of household tasks means that women and girls are usually in charge of washing clothes and collecting water and thus more exposed to contaminated water, and therefore schistosomiasis and other water-borne infections [68]. However, there can be little debate regarding the impact of schistosomiasis infection on pregnant women, with urogenital schistosomiasis [69, 70] being a common gynaecologic condition among African women [71], a significant cause of pregnancy complications [66, 72, 73] with an increased risk of HIV transmission in women due to the presence of genital lesions [74, 75]. Pregnant women also suffer from hookworm-associated anaemia and dengue in pregnancy [76, 77]. More recently, the impact of Zika virus causing congenital birth defects, including microcephaly and other neurological sequelae and Guillain-Barré syndrome has emerged [78]. While Zika is not yet formally recognized as an NTD, it has already being discussed in that framework [79]. Women are also susceptible to mother-to-child transmission of Chagas disease [80], among other conditions [81]. There is also some evidence that co-infection with *Plasmodium falciparum* and helminths affect predominantly school-age children and pregnant women. These groups, which have the highest risk of anaemia, would benefit from an integrated approach to malaria and helminth control [82].

Finally, women’s disproportionate poverty, illiteracy, lack of education, land ownership and political voice act as barriers to health-seeking, and may increase their exposure to NTDs and their sequelae [68]. In this context, the empowerment of women becomes a crucial aspect of the fight against NTDs, and interventions focused on NTD control and elimination offer important opportunities for improving the health and rights of girls and women in the populations affected [66].

With regard to specific NTD interventions, it has been demonstrated that treatment of women for hookworm in the second and third trimester increases infant survival in hookworm endemic areas [83]. One study, examining the association between anthelminthic treatment with the drug albendazole and maternal anaemia, birth weight, and infant mortality, revealed that women given albendazole in the second trimester had a lower rate of severe anaemia during the third trimester. Birth weight of infants of women who had received two doses of albendazole rose by 59 g, and infant mortality at 6 months fell by 41%. Thus, it appears that antenatal anthelminthic treatment is effective at reducing maternal anaemia and improving birth weight and infant survival in hookworm-endemic regions. In addition praziquantel used in schistosomiasis MDA can also be safely used in pregnancy thereby improving anaemia status of infected women [83].

There is also some evidence of proportionately greater benefit for women of MDA. For example, coverage surveys from 37 countries, showed a gender ratio slightly in favour of women (between 0.96 and 1.17) [84], an observation confirmed by data from Uganda [85]. Qualitative data collected from district health workers, community leaders, community medicine distributors and community members suggested that socio-behavioural and structural barriers to treatment access may be present for both genders. However, where Community Directed Distribution (CDD) was introduced, an increasing number of women attended meetings, spoke out and were then selected as community implementers. Over time, women also became more outspoken, participated more actively, and demanded to be assigned responsibilities. Community-based organizations, including women’s groups, became more involved in the process. For instance, in one Nigerian site, the market women’s association now plays an active role in community health activities [86]. Many community members have also reported that women were more committed, persuasive and more patient than men in the distribution of ivermectin [87].

### SDG 6 Ensure access to water and sanitation for all

Progress on water and sanitation has been remarkable over recent decades, with an estimated 2.1 billion people gaining access to an improved water source since 1990. According to the most recent data, nearly 90% of the global population has access to an improved water source, which means water that is either piped into a dwelling, plot or yard, obtained from a public tap, or well, or collected from a protected spring or rainwater [88]. Unfortunately, this still means that around 633 million people do not have access to an improved drinking water source, while access does not mean that the water being collected is necessarily clean. A recent literature review reported that in 38% of 191 studies, over a quarter of samples from improved sources contained faecal contamination [89]. Meanwhile, an estimated 40% of people around the world are affected by water scarcity, a number that is projected to increase with the rise of average global temperatures. Progress on sanitation has been even less impressive, with 36% of the world’s population, or nearly 2.5 billion people, lacking access to improved sanitation facilities putting them at risk of enteric diseases including dysentery, cholera, typhoid, schistosomiasis, and intestinal worms. [88]

Ensuring universal access to safe, reliable and affordable drinking water by 2030 requires investment in infrastructure to support the provision of sanitation facilities, and investments in efforts to support behavioural change, discouraging, in particular, the widespread practice of open defecation. It will also require protecting and restoring water-related ecosystems such as forests, mountains, wetlands. Greater international cooperation is also needed to encourage water efficiency and support of treatment technologies in developing countries [90].

NTD programmes and initiatives have a privileged role to play in achieving those goals because of the prime importance of water and intermediate hosts in the lifecycle of many pathogens. NTDs thrive where water and sanitation are inadequate. For example, water contaminated with faeces and urine can contain worm eggs that contaminate surface water and lead to transmission of schistosomiasis. These can come from the faeces and urine of human and reservoir hosts, such as cows and buffalos, making it important to protect freshwater from animals and their waste. Poorly-constructed latrines facilitate the breeding of *Culex* mosquitoes, causing great nuisance as well as being efficient vectors of lymphatic filariasis and some viruses [91]. Replacement of pit latrines with modern toilets would contribute to a reduction in nuisance biting and transmission reduction [92]. Similarly, *Aedes aegypti*, the major vector of arboviruses, could be reduced by environmental management. This urban species breeds in many small uncontaminated water bodies [93]. In many cases where piped water is lacking or unreliable, water-storage containers become major larval habitats for *Ae. aegypti* and *Ae. albopictus* mosquitoes which transmit dengue, Zika, yellow fever and chikungunya viruses to humans.

Meanwhile, access to clean water is essential for tackling some diseases. For example, trachoma, a leading cause of preventable blindness, is caused by a bacterial (*Chlamydia trachomatis*) infection transmitted through contact with eye-seeking flies (*Musca sorbens*, a fly closely associated with breeding in human faeces), dirty fingers and fomites. Facial cleanliness and environmental improvement are primary prevention components of WHO’s SAFE strategy for trachoma elimination [94], requiring clean water for face washing to remove eye discharges. Unfortunately, the people affected by NTDs are often stigmatized and can be excluded from accessing water and sanitation facilities, increasing their risk of poverty and severe illness [56].

Water, sanitation and hygiene (WASH) interventions are essential components in preventing many NTDs, as attested by individual studies, reports and systematic reviews. The work of the Guinea Worm Eradication Programme’s (GWEP) in assisting in the provision of increased access to safe water through provision of bore holes and protected wells is notable in this regard. [95] Other studies conclude the development and management of water resources is vital in schistosomiasis control [96] and that individual household latrines are associated with lower STH infection versus shared sanitation facilities [97].

Various systematic reviews have been published with findings that include: safe water being associated with significantly reduced chance of *Schistosoma* infection and access to adequate sanitation being associated with significantly lower odds of infection with both *S. mansoni* and *S. haematobium* [98]; better hygiene in children being associated with lower odds of developing trachoma and access to sanitation being associated with 15% lower odds of active trachoma and 33% lower odds of *C. trachomatis* infection of the eyes [99]; and WASH access and practices being associated with 33–70% lower odds of STH infection (people who wash their hands after defecating are less than half as likely to be infected as those who did not) [100].

Reflecting the cross-sectoral nature of the WASH challenge, and the fact that the WASH component of the NTD strategy has not received attention in line with its importance, in August 2015 the WHO launched a global strategy and action plan to integrate WASH with other public health interventions [101]. The expectation is that a more integrated approach to NTDs and WASH efforts will increase efficiencies and enhance sustainability. It will also ensure that investments in WASH reach those most in need. Achieving universal access to WASH requires a focus on the poorest and hardest to reach, the same groups most affected by NTDs. However, the target date for the WHO NTD roadmap is 2020, 10 years earlier than WASH, (the WASH sector is focused on the SDG target of universal access to basic WASH in communities, schools and healthcare facilities by 2030). Integration may thus add impetus to the need for progress on the WASH agenda for the most vulnerable. Only the achievement of the WASH targets will ensure that sustained reduction of transmission of those NTDs associated with water and sanitation will be achieved. Thus, progress on certain NTDs can also serve as a proxy for equity and effective targeting of WASH programmes. The joint strategy has the potential to make an important contribution to global efforts to achieve the SDGs, most notably in regard to the achievement of UHC whilst addressing some of the key determinants of human health.

### SDG 7. Ensure access to affordable, reliable, sustainable and modern energy for all

According to the United Nation’s Development Programme the number of people with access to electricity increased by 1.7 billion between 1990 and 2010. While this represents an impressive advance in development, since it is based almost exclusively on fossil fuels it will increase greenhouse gas emissions, and has the potential to exacerbate climate change. Thus the 2030 Agenda calls for a switch towards renewable energy such as solar, wind and thermal, and for the adoption of cost-effective standards for a wider range of technologies. Expanding infrastructure and upgrading technology to provide clean energy sources in all developing countries is seen as a crucial goal that can both encourage growth and help the environment.

In certain areas there is a significant degree of alignment between NTD and sustainable energy agendas. For example, a move towards clean energy technologies such as biogas systems, which produce methane from human and animal waste, has the potential to provide immediate benefits for the control of NTDs. This was borne out by an assessment of the parasitic disease and energy benefits of biogas systems in Sichuan Province, China, where the burning of effluent not only generated energy in a sustainable way, but also inactivated the schistosomiasis eggs contained within [102]. This highlights ways in which the public health sector can leverage the proliferation of rural energy projects as a part of infectious disease control.

Sustainable energy strategies that integrate rural energy needs and sanitation offer considerable promise for the long-term control of parasitic diseases, while simultaneously reducing energy costs and improving quality of life. Government policies can enhance the financial viability of such strategies by introducing fiscal incentives for joint sanitation/sustainable energy projects, along with their associated public outreach and education programs. [103] Meanwhile, zoonotic NTD control programmes can contribute to such projects by targeting those communities where the returns from biofuel generation are expected to be largest and in ensuring safe access to reliable sources of biomass.

It can also be argued that efforts to mitigate the impact of NTDs enhances the feasibility and sustainability of hydroelectricity projects. For example, it has been shown that the construction of large dams has led to increases in human schistosomiasis in China, Cote d’Ivoire, Egypt, Ethiopia, Senegal [104], and Sudan, amongst others [105–107]. Dam spillways are known to serve as breeding sites for blackflies [108], and the construction of dams can lead to complex changes in habitat, which in addition to movement of non-immune populations and snails into the area can lead to transmission hotspots [109]. Evidence for beneficial inter-sectoral collaboration includes a study examining the impact of dam construction in Senegal and Mali, which showed the beneficial impact of control of water-related NTDs, including control of snails and mosquitoes [109]. When initiating sustainable energy infrastructure, the existence of good control interventions for NTDs can ensure that the negative impact on health caused by NTDs will be mitigated.

### SDG 8. Promote inclusive and sustainable economic growth, employment and decent work for all

Over the past 25 years, the number of workers living in extreme poverty has declined steeply, despite the aftershocks of the 2008–2009 financial and economic crisis. However, sluggish global economic growth, widening economic inequalities and employment that is not expanding fast enough to keep up with the growing labour force are significant sources of concern. According to the International Labour Organization, more than 204 million people were unemployed in 2015. The SDGs aim to encourage sustained economic growth by achieving higher levels of productivity and through technological innovation. Promoting policies that encourage entrepreneurship and job creation are key to economic growth, as are effective measures to eradicate forced labour, slavery and human trafficking. With these targets in mind, the goal is to achieve full and productive employment, and rewarding work, for all women and men by 2030.

The economic effect of NTDs, taken together with the social and psychological burden they impose is enormous [110, 111]. The financial and economic costs of treatment and control of NTDs are generally underestimated because of the tendency to focus on the costs to the health care system itself, and in particular the costs faced by government-funded health facilities. Other costs relate to lost productivity [112]. A study of tea pickers in Bangladesh living with three worm infections (*Ascaris lumbricoides, Trichuris trichiura*, and hookworms), showed a negative association between the intensity of helminth infections (eggs and faeces) and productivity [113]. Losses can also accrue because of households and businesses adapting their activities in response to the NTD burden. So-called coping strategies can lead to decreased savings and investment, lost capital and purchasing power, and inefficient labour substitution [114].

Clearly, a healthy workforce is critical for economic development. The disabilities, disfigurement and debilitating effects, including mental health impacts [27, 115], of NTDs, as well as secondary impacts such as exclusion and prejudice, prevent adults from working, providing for their families and contributing to the economic development of their countries. As noted in regard to SDG 1, at the household level, this can trap generations in a cycle of increased medical costs, poverty and disease; at the macro level it results in significant economic losses for countries. Evidence for this includes a study of lymphatic filariasis in India which showed that every year $842 million are lost due to treatment costs and reduced working time, equivalent to $2 per person resident in endemic areas [116]. The global economic burden of rabies is about $8.6 billion dollars lost [117], while the economic burden of dengue in the Americas is $2.1 billion dollars lost. [118] NTD programmes can have a significant impact on individuals’ employment prospects, and by extension, national productivity. Evidence for this includes a study conducted in the southern USA which estimated that childhood treatment for hookworm increased future wages by approximately 40% [119], and the above-mentioned study covering the long-term impact of deworming in Kenya, which showed that future earnings are up to 29% higher for children targeted by the campaign, while hours worked increased by 12% and work days lost to illness decreased by a third. A study undertaken in Egypt, showed that workers with schistosomiasis increased productivity if they could access early detection and treatment [120]. Thus, interventions to control NTDs promise significant economic pay-offs outside the health sector in agricultural productivity and educational benefit, and should be considered as investments in human capital and poverty reduction [121]. A recent study has estimated that the impact of MDA during the past 13 years prevented or cured 96 million cases of lymphatic filariasis, despite 36 million cases of hydrocoele and lymphoedema remaining [122]. However, the economic benefits estimated to have accrued during the first 8 years of the programme exceeded $24 billion. A 2016 study on health gains from NTD programmes has estimated if targets are met for nine NTDs, 600 million Disability Adjusted Life Years (DALYs) would be averted, which can be extrapolated to 2030, due largely to the decline in morbidity of NTDs treated by preventive chemotherapy [123]. However, this figure does not include gains in subtle morbidities through improved cognitive development and mental health and the analysis was restricted to only nine NTDs. This suggests that overall gains in DALYs from all NTD programmes would be substantially greater than the 600 million DALY figure, thereby having a significant impact on economic growth of affected communities.

### SDG 9. Build resilient infrastructure, promote sustainable industrialization and foster innovation

Sustained investment in infrastructure and innovation are crucial drivers of economic growth and development. SDG 9 targets the development of quality, reliable, sustainable and resilient infrastructure, including regional and trans-border infrastructure, to support economic development and human well-being, with a focus on affordable and equitable access for all. Key to achieving this will be significantly raising industry’s share of employment and Gross Domestic Product (GDP), in line with national circumstances, an overall target that includes doubling the share of industry in the employment and GDP of the least developed countries.

The drive to end NTDs has made a considerable contribution to logistics infrastructure generally and particularly at the community level. MDA has required the development of, and investment in, supply chains, including transport and storage infrastructure and the clinics ensuring the availability of donated medicines [86, 124]. The “Supply Chain Forum”, a landmark private and public partnership consortium, has ensured that endemic countries are able to efficiently request and receive supplies for NTD interventions. This includes the development of a tool to streamline supply chain management, the creation of a dedicated DHL control tower to facilitate clearance through customs and statistical tools to forecast demand [125]. NTD programmes and initiatives have also enhanced the capacity of laboratories to diagnose diseases [126], conduct independent clinical research [127] and has led to the construction of roads, markets, schools and clinics [128].

Health R&D encompasses the WHO strategy for research and innovation in health and aims to develop technologies (medicines, diagnostics, medical devices, vaccines, etc.) for diseases, including the NTDs, that disproportionately affect developing countries [129]. The Visceral Leishmaniasis Global R&D and Access Initiative, for example, addresses some of the critical R&D gaps in order to provide supporting tools to meet the WHO elimination goals for visceral leishmaniasis (VL). These demonstration projects as part of the aim to end the epidemic of NTDs, can serve as an example of how innovation can be fostered beyond the NTDs.

In order to address the under-representation of African scientists in global priority setting for research on NTDs, five European foundations (Volkswagen, Gulbenkian, Mérieux, Nuffield and Cariplo foundations) formed the European Foundation Initiative for African Research into Neglected Tropical Diseases (EFINTD) in 2008 [130]. EFINTD has strengthened research capacity in NTDs by empowering young African researchers who are based in their home countries to perform research on various NTDs [131] and has created the African Network for NTDs (ARNTD).

### SDG 10. Reduce inequality within and among countries

Increased global productivity, driven largely by productivity gains in Asia, has contributed to the reduction of global poverty levels. However, inequality persists, and large disparities remain in access to health and education services and other assets. Moreover, while income inequality between countries has been reduced, inequality within countries has risen. There is a growing consensus around the idea that economic growth in itself is not sufficient to reduce poverty, if it does not benefit everyone, and does not involve the economic, social and environmental dimensions of sustainable development. Central to progress in this area (as with health) is the development of a rights-based policy that addresses the needs of disadvantaged and marginalized populations. Specifically, SDG 10 calls for sustained income growth for the bottom 40% of the population at a rate higher than the national average (to be achieved by 2030). This neglected section of society is also most affected by NTDs. In Nigeria, for example, more than 60% of school-aged children revealed inequalities in NTD prevalence across socioeconomic groups, with the poor shouldering the greatest burden [132]. In Honduras, being in a family from higher socio-economic status was protective against infections by roundworm (*Ascaris*) and whipworm (*Trichuris*) [133]. A study carried out in Nigeria in 2009 found that the prevalence of ascariasis among children ranges from 10% when both parents have at least primary education, to 96% when neither parent does [134]. A 2015 case-control study using asset-based analysis undertaken in Ethiopia found not only that trichiasis cases are more likely to occur in poorer households but that people living with trichiasis were less likely to participate in economically productive activities regardless of visual impairment and other health problems [135].

Ending NTDs would contribute to reducing these inequalities and move closer to achieving the targets of SDG 10. At the same time, SDG 10 also calls for efforts to empower and promote the social, economic and political inclusion of all, irrespective of age, sex, disability, race, ethnicity, origin, religion or economic or other status. Here, too, NTD programmes and initiatives have an important part to play in reducing discrimination and the exclusion and stigmatisation of those living with NTDs.

### SDG 11. Make cities inclusive, safe, resilient and sustainable

Over half of the world’s population now live in urban areas, and by 2050, it is expected that cities will accommodate 6.5 billion people, two-thirds of humanity. The growth of cities in the developing world, driven in part by increasing rural to urban migration, has led to the astonishing development of mega-cities. In 1990, there were ten mega-cities with 10 million inhabitants or more. In 2014, there were 28 mega cities, home to 453 million people. Given the strains already apparent in urban centres, it is clear that sustainable development cannot be achieved without massively transforming the way we build and manage urban spaces. To make such cities safe and sustainable will require access to safe and affordable housing, which will in turn require upgrading slum settlements. It will also involve investment in mass transport systems, the creation of green spaces, and urban planning and management that is both participatory and inclusive.

Often thought of as a concern solely for remote rural areas, several NTDs are rooted in the urban environment. This is partly because of the conditions in which the urban poor often live and partly because of the affinity for urban areas of certain NTD vectors. The recent Zika outbreak was a wake-up call to the world, emphasizing the necessity to improve the control of vector-borne diseases, particularly in urban settings, which remain vulnerable to the transmission of *Aedes*-borne diseases. *Ae. aegypti*, which transmits dengue, Zika, chikungunya, and yellow fever, thrive in towns and cities throughout the tropics and sub-tropics. Urban environments provide ideal larval habitats for this mosquito including water storage containers, tyres, guttering, and the plethora of plastic containers discarded as waste. For diseases, such as leishmaniasis, normally associated with rural environments, transmission in urban and peri-urban settings has been reported in Brazil [136], Sudan (Khartoum) and in Burkina Faso (Ougadougou) and in Europe [137] leading to an outbreak in a major European capital (Madrid) [138]. Movement of people and mosquitoes will also increase the risk of establishing NTDs in cities previously not affected. The 2013 outbreak of chikungunya in the Caribbean, for example, led to a seven-fold increase of imported infections to Spanish cities where the vector was present, thereby posing a threat of autochthonous transmission [139].

The global incidence of dengue has increased dramatically in recent decades, partly as a result of urban growth, with the result that roughly half of the world’s population is now at risk of infection [140]. Dengue thrives in urban slums, as indicated by a study based on community-based surveillance in a slum in Salvador, Brazil which found 22% of febrile patients with evidence of dengue infection. The study also suggested that socioeconomic development could potentially mitigate risk factors for both dengue and non-dengue febrile cases, and found that residential proximity to a health care facility was associated with improved case detection [141].

WHO guidelines on preventing or reducing dengue virus transmission focus on control of the mosquito vectors or interruption of human–vector contact. Activities to control transmission are directed at removing the habitats of the immature larval stages in the household and immediate vicinity, as well as other settings where human–vector contact occurs (e.g., schools, hospitals and workplaces), unless there is sound evidence that *Ae. albopictus* or other mosquito species are the local vectors of dengue. The provision of reliable piped water, which prevents people having to store water and creates potential larval habitats, and efficient domestic waste management, will lead to fewer larval habitats for *Ae. aegypti* and house screening would help reduce the transmission of *Aedes*-borne diseases. More emphasis is being given to community-based vector control strategies that include environmental management [142].

The triatomine bug, a vector for Chagas disease, is another cause of concern as it is associated with poor housing in urban areas [143]. This despite, previous successes in the Southern Cone countries of the Americas in controlling domestic transmission of Chagas’ diseases by *Triatoma infestans* by indoor residual spraying in urban settings [144, 145]. The control of Chagas’ disease was based on insecticide spraying to eliminate household infestation. Because of the focus on domestic transmission, there has been a tendency to neglect peri-domestic transmission. Housing improvement, to reduce the number of sites within houses where vectors can live and breed (cracks in walls, poor roofing materials, behind pictures) is also a key part of Chagas disease control, usually as a part of integrated approaches that combine spraying, environmental organization and improvement, health education and community mobilization. As with so many NTD-related endeavours, community participation and ownership is crucial [146].

*Aedes*-borne diseases like dengue, Zika, yellow fever and chikungunya, and Chagas control initiatives and targets are closely aligned with the targets set out in SDG 11. The effective prevention of NTDs addresses many of the social factors that produce unequal health outcomes among slum residents, in addition to improving sanitation through integrated NTD-WASH interventions. Environmental health, in particular, recognising the need to control vectors should be incorporated into future planning within cities. In Spain, for example, an unusual urban outbreak of leishmaniasis was attributed to a conversion of agricultural land to urban parkland, which may have brought a wild transmission cycle into contact with people [138], and could have been prevented if vector borne diseases were considered during the planning stage.

### SDG 12. Ensure sustainable consumption and production patterns

Achieving economic growth and sustainable development will require a change in the way we produce and consume goods and resources. The efficient management of shared natural resources, and sustainable management of waste disposal, including toxic waste, are core targets for SDG 12. Halving per capita global food waste at the retailer and consumer levels, for example, will create more efficient production and supply chains, by reducing food losses along production and supply chains, including post-harvest losses. In future, efforts will also focus on encouraging industries, businesses and consumers to recycle and reduce waste.

Of the eleven specific targets for SDG 12, NTD efforts have a specific bearing on target 4 which calls for the achievement, by 2020, of environmentally sound management of chemicals and all wastes throughout their life cycle, in accordance with agreed international frameworks, and significantly reduce their release to air, water and soil in order to minimize their adverse impacts on human health and the environment. Efforts to end NTDs align with this target in two important ways, the first being the responsible production, use and disposal of pesticides.

Insecticide resistance arising from overreliance on specific insecticides used in long-lasting insecticidal nets and in many indoor residual spraying programmes, is a major cause of concern for NTD and malaria programmes [147–149]. WHO’s work in this area focuses on the WHO Pesticide Evaluation Scheme (WHOPES), a cross-cutting programme based in the Vector Ecology and Management Unit of the Department of Control of NTDs, which supports United Nations agencies and WHO programmes, and assists Member States with vector control programme implementation [150]. In 1997 WHOPES established the Global Collaboration for the Development of Pesticides for Public Health (GCDPP) in response to the need to stimulate the development of alternative insecticides and application technologies [150]. GCDPP meetings bring together major stakeholders in pesticide product development and the sound management of pesticides, to discuss and strengthen collaboration on critical vector control issues.

Efforts to end NTDs also align with target 4 in relation to waste disposal, focusing on the sustainable production and recycling of rubber tyres, plastic bottles and other containers as a part of vector control efforts designed to halt the global spread of *Aedes* mosquitos [151, 152]. *Ae. aegypti* proliferates in purposely-filled household containers such as those used for domestic water storage and for decorative plants, but also in a multiplicity of rain-filled habitats – including used tyres, discarded food and beverage containers, blocked gutters and buildings under construction. Typically, the mosquitoes do not fly far, most remaining within 100 m of where they emerged [153]. A study of porcelain and plastic household wastes serving as larval habitats of vectors of *Aedes*-borne diseases in rural and urban areas around Kolkata, India, found that porcelain and plastic wastes were more productive (i.e., more larvae developed in them) in urban areas compared to rural areas. The risk of dengue epidemics was expected to increase if waste generation continued without appropriate measures being applied to limit the addition of such waste to the environment. The study suggested that increase use of biodegradable containers might reduce the problem of waste and mosquito production [154].

### SDG 13. Take urgent action to combat climate change and its impacts

SDG 13 calls for urgent collective action on climate change and its impacts, and focuses on five targets, three of which have particular relevance for NTD programmes. These are: target one, on strengthening the resilience and adaptive capacity to climate-related hazards and natural disasters in all countries; target three on improving education, awareness-raising and human and institutional capacity on climate change mitigation, adaptation, impact reduction and early warning; and target five on promoting mechanisms for raising capacity for effective climate change-related planning and management in least developed countries and small island developing States, including focusing on women, youth and local and marginalized communities.

Strengthening the resilience and adaptive capacity to climate-related hazards depends in part on an understanding of the links between climate change and the epidemiology of certain NTDs. The fact that climate change is happening and is likely to expand the geographical distribution of several vector borne infections is well established [155]. The spread of malaria is, of course, a major concern, but in the context of the NTDs, attention is focused on the spread of dengue, for which there is increasing evidence of an association between epidemics and temperature, rainfall and relative humidity [156]. In response to drought, for example, water collection frequently occurs resulting in new breeding habitats for vectors [157]. This is further exemplified by the northward spread of the Chagas disease [158] and leishmaniasis [159] vectors in the USA and Europe, respectively. Similarly, mean water temperatures are expected to increase in the south of Europe which will enhance the capacity of snails to overwinter, leading to more schistosomiasis outbreaks as seen in Corsica (France) [26].

The effectiveness of future responses to these diseases requires an understanding of the changing dynamics of the pathogen, host (human and/or reservoir animal) and vector as well as the environmental dynamics which result from climate change. Each species responds to environmental changes differently, and in order to predict the movement of disease through ecosystems, we have to rely on expertise from the fields of veterinary, medical, and public health in line with the One Health strategy [160]. Modelling and surveillance of human and vector behaviour can also help us better understand the environmental and climatic factors driving diseases and help counteract potential impacts [161, 162].

Target three, focuses on awareness and education; NTD programmes raise awareness and increase knowledge among populations, public health practitioners, and policy makers about vector-transmitted infections and their relationship to disease which serves as the basis for prevention strategies such as personal protection, improved hygiene and reducing exposure to vectors or intermediate hosts (snails and schistosomiasis/*Cyclops* and Guinea worm).

### SDG 14. Conserve and sustainably use the oceans, seas and marine resources

Over three billion people depend on marine and coastal biodiversity for their livelihoods. However, decades of overfishing and pollution of the oceans has brought marine environments and fish stocks to the point of collapse. It estimated that 30% of the world’s fish stocks are over-exploited, well above a level to produce sustainable yields. Marine pollution, an overwhelming majority of which comes from land-based sources, is reaching alarming levels, with an average of 13 000 pieces of plastic litter to be found on every square kilometre of ocean. Oceans are also changing as a result of greenhouse gas emissions driven by industrialisation as they absorb about 30% of the carbon dioxide produced by humans and hence are becoming increasingly acid. There has been a 26% rise in ocean acidification since the beginning of the industrial revolution. Similarly important and at-risk are fresh-water bodies, which provide the equivalent of all dietary animal protein for 158 million people, particularly in poor and undernourished populations [163]. The Sustainable Development Goals create a framework to sustainably manage and protect marine and coastal ecosystems from land-based pollution, as well as address the impacts of ocean acidification. Enhancing conservation and the sustainable use of ocean-based resources through international law will also help mitigate some of the challenges facing our oceans.

In addition to water pollution and overfishing, fresh water fish populations can be affected by diseases. Fish-borne zoonotic trematodes (FZT) are important emerging and re-emerging pathogens causing liver and intestinal fluke diseases in humans [164]. It is estimated that 750 million people worldwide are at risk of infection [165] with more than 46 million people infected [166]. Parasitic infection also impacts fish, resulting in reduced fitness, which impacts on the food web and therefore also on the entire marine ecosystem [167]. Trematode parasite larvae in water bodies, which use snails as their intermediate host, infect and mature within fish or crustacea. Humans are then infected when they eat these fish either raw, inadequately cooked, dried, salted or pickled, and re-introduce these parasites into the water via faeces [168].

As such, NTD interventions will have an impact on several trematode diseases. Where human faeces are disposed of directly into water (sometimes to feed fish stocks), treatment of infected individuals can help break the cycle of transmission and improve the health of fish populations and safety of small-scale fish production [169, 170]. Secondly, education and provision of Water Sanitation and Hygiene (WASH) coverage, part of the joint WASH-NTD strategy, would prevent open defecation and reduce infections [101].

Even though the impact of FZT on aquatic ecosystems is limited in comparison to pollution and overfishing, addressing the burden of NTDs will also contribute to achieving some of SDG14 targets.

### SDG 15. Sustainably manage forests, combat desertification, halt and reverse land degradation, halt biodiversity loss

In a list of extremely broad and ambitious goals, SDG 15 is one of the broadest and most ambitious, addressing the colossal challenges such as land degradation and biodiversity loss. The specific references to forest management, and the issue of desertification offers some scope for focus. The impacts of NTDs, and NTD responses on these targets is particular rather than general, but they are nevertheless significant, notably with regard to reducing the impact of invasive parasites and pests on ecosystems [171].

One of the key drivers of the NTD epidemic in recent years is the expansion of industrial-scale plantations –monocultures- that are actively managed for the commercial production of commodities from lumber to latex. The unprecedented expansion in global trade in recent decades [172] has encouraged the expansion of rubber plantations in South East Asia which provide ideal habitats for the mosquito vectors of malaria, dengue, and chikungunya [171]. Similarly, illegal logging and other plantation cultures including teak, palm oil, pine forest and eucalyptus have created ideal environment for transmission of vector borne infections [173]. Often tended by migrant workers, frequently non immune groups with no previous exposure to these infections, they also act as repositories for pathogens brought in from outside, most worryingly artemisinin-resistant malaria parasites [174]. The close proximity of rubber plantations to natural forest also increases the threat from zoonoses, where new vector-borne pathogens spill over from wild animals into humans [175]. Previous reintroduction of zoonotic disease in ecosystems has led to loss of wildlife and even the risk of extinction of some species, such as wild dogs in Africa due to rabies [176].

There is, therefore, a need to scale up surveillance of vectors and diseases as well as access to health care for plantation workers. Achieving this will require an inter-sectoral approach with strong collaboration between the health sector, the forest industries and local communities. By addressing vector-borne NTDs, particularly in migrant workers, the introduction of alien vectors and arboviruses into these ecosystems can be prevented [177].

Another area of concern for NTD programmes that has direct relevance for SDG 15 is the degradation of land and soil as a result of unsafe sanitation arrangements and practices such as open defecation. Such degradation is a significant driver of ascariasis, trichuriasis and hookworm disease, all of which are transmitted via soil and other media that are contaminated with excreta containing infective eggs or larvae. Transmission typically takes place near the home, or in a communal area with inadequate sanitation facilities and occurs when infective eggs are ingested, and in the case of hookworm disease, also when infective larvae penetrate the skin [178]. Transmission does not occur from person-to-person or from contact with fresh faeces because it takes time for the parasite to develop. For this reason these nematode infections are considered to be entirely attributable to the environment, and occur because of a lack of excreta management and inadequate hygiene practices [179].

NTD programme responses have focused on deworming which has been shown to reduce environmental contamination with soil transmitted helminth eggs [180]. However, rapid re-infection occurs in areas where hygiene, access to clean water and sanitation are inadequate [181]. Therefore, WASH interventions are a vital a component of any response. The community-led total sanitation approach that is a core focus of UNICEF’s sanitation work has been successful in several countries, notable among them, Ethiopia. A key aspect of the approach is effectively communicating to communities the risks of open defecation. However, behaviour change is not possible without the latrines needed to accommodate it. UNICEF works in conjunction with the Ethiopian government to engage the private sector by marketing sanitation throughout the country. The push to upgrade the country’s toilets dates back to 2003 in the Southern Nations Nationalities and Peoples Region (SNNPR) of Ethiopia, where it became compulsory for each household to have and use a latrine. Today, the SNNPR reports 75% access to sanitation, the highest of any region in Ethiopia. In the country, as a whole, access to safe sanitation has risen from 7% in 2000 to 60% today [182].

### SDG 16. Promote just, peaceful and inclusive societies

Ensuring peace, stability, human rights and effective governance based on the rule of law is a prerequisite for sustainable development. SDG 16 aims to significantly reduce all forms of violence, and work with governments and communities to find lasting solutions to conflict and insecurity. Strengthening the rule of law and promoting human rights is key to this process, as is reducing the flow of illicit arms and strengthening the participation of developing countries in the institutions of global governance.

Advocacy for the elimination of NTDs can also promote peace. Importantly, the Guinea worm cease fire negotiated by President Jimmy Carter in South Sudan not only enabled progress in Guinea worm eradication to be maintained, but appeased warring factions, albeit for a short time [183]. Such peace deals will become more important as countries apply for the certification of the absence of Guinea worm transmission in the remaining countries yet to be certified. However, a major challenge will be to achieve access to areas where there are security problems, such as in Kidal, Mali and West Darfur [184]. Similarly, the effects of conflict on leishmaniasis are well documented, where the current refugee crisis has led to unprecedented number of new cases of the cutaneous form of the disease [185–187].

Interventions against NTDs are unlikely to be prioritised in these situations where humanitarian needs are the first concern of agencies involved in the many geopolitical crises. This is notably the case in regard to reducing the fear that drives institutional discrimination against NTD-related disability. People affected by NTDs are frequently the target of social stigmatization. Leprosy is an obvious example [188], and has been a major focus of stigma studies in the past. However, there are many other NTDs for which stigma is an important consideration, including onchocerciasis [189], lymphatic filariasis [115], Buruli ulcer [190], leishmaniasis [191], schistosomiasis [192] and Chagas disease [193]. The psycho-social aspects of stigmatisation associated with disfiguring NTDs is well documented, but stigma is also an important social determinant of the effectiveness of disease control because of its impact on help-seeking and treatment adherence. Furthermore, it has been shown that stigma influences political commitment to NTD control [56, 194].

Efforts to control NTDs include initiatives to encourage social inclusion and may sensitize local populations to risks, reducing fear and stigma that are manifested also by local institutions, in spite of non-discrimination laws. A good example of this was the launching of a powerful and broad-based advertising campaign to change the public image of leprosy by the Sri Lankan Ministry of Health, assisted by international support, in 1990. By portraying leprosy as just another treatable disease, the campaign hoped to encourage people with suspicious lesions to come forward for early diagnosis and cure free of charge. The campaign strongly reduced the stigma attached to leprosy, enabling a shift from reactions of fear and loathing to understanding and compassion, and for those living with disease, a shift from despair to hope for a cure. As a result of this and other interventions, the public health problem of leprosy has been eliminated in Sri Lanka since 1996.

Research suggests that the most effective approach to this issue may be joint interventions rather than initiatives focused on one disease at a time. A systematic review of 52 articles which provided evidence on stigma found that commonalities in the reasons for and nature of the stigma applying to all of the diseases considered suggested that a joint approach to reduce stigmatization might be feasible. The same study suggested that lessons from leprosy and other stigmatizing health conditions can be used to plan such joint approaches [194].

### SDG 17 Revitalize the global partnership for sustainable development

As noted at the outset, the SDGs can only be realized with a strong commitment to global partnership and cooperation. A successful sustainable development agenda requires partnerships between governments, the private sector and civil society. These inclusive partnerships built upon principles and values, a shared vision, and shared goals that place people and the planet at the centre, are needed at the global, regional, national and local level. SDG 17 calls for urgent action to mobilize, redirect and unlock the transformative power of trillions of dollars of private resources to deliver on sustainable development objectives.

The NTD agenda has been characterised by strong global partnerships from the beginning. Under the guidance or leadership of WHO, these have proven key to tackling the diseases effectively and have helped to mobilise resources [2]. Examples abound [195], dating back to the early 1990s with the exception of International Federation of Anti-*Leprosy* Associations (*ILEP*) which was founded in 1966. Relatively recent additions include the NTD NGDO Network which was established in October 2009, and provides a global forum for non-governmental development organizations (NGDOs) and a wide range of other partners, and Uniting to Combat NTDs, a collective of invested, interested and dedicated partners, working to fulfil the London Declaration on NTDs (2012).

Such partnerships have brought together a broad range of actors, including governments and other stakeholders in endemic countries, international agencies, pharmaceutical companies, international non-governmental organizations, academia, civil society and UN agencies [4]. Many of these partners have come together as a result of more integrated approaches to prevention and control, which have coalesced around the WHO’s Global Plan to combat neglected tropical diseases, 2008 – 2015, and the Roadmap for Implementation.

Inspired by the 2020 Roadmap, a group of high-profile stakeholders including bilateral donors, the World Bank, the Bill and Melinda Gates Foundation, and several pharmaceutical companies, came together under the London Declaration of 2012, pledging to provide the resources necessary for implementation. A cornerstone of the London Declaration is the contribution of donated drugs from pharmaceutical companies. Over 5.5 billion tablets have been donated providing 3.5 billion treatments since the launch of the London Declaration in 2012. In 2014, 1.45 billion treatments were made available to endemic countries, representing a 36% increase since 2011.

Public–private partnerships (PPPs) in NTDs have been key [196], bringing together NGOs, who, working in partnership with governments, have played a critical role in advocacy, resource mobilisation and implementation. Their technical assistance has helped governments to develop community-based methods of drug distribution, ensuring that ‘essential drugs’ reach those most in need [196]. PPPs have also been crucial in driving costs down allowing NTDs to be treated in an extremely cost-effective way. It has been estimated that treating one person for all seven major NTDs costs approximately 50 cents per year, which can be even lower in Asian countries [197–199].

In many ways ending NTDs exemplify the potential of public private partnership and domestic financing. The global effort to control and eliminate NTDs is one of the largest public health initiatives ever. In 2015 alone, pharmaceutical companies donated an estimated 2.4 billion tablets, enough for 1.5 billion treatments to prevent and treat NTDs.

## Conclusions

NTDs and the responses developed for their control or elimination, are woven into the fabric of the 2030 Agenda. In this scoping review we have shown how NTD programmes and initiatives have implications for multiple goals (see Table 1). This is true, for example, of preventive chemotherapy, which, as demonstrated here, has a bearing on poverty, hunger, education, employment and equality, to name the SDGs’ most directly impacted. The provision of clean water and sanitation as part of integrated WASH responses against NTDs that thrive where these are inadequate, have impact beyond the interruption of disease transmission, and can elevate the quality of life of those affected. We have shown how community-led distribution of medicines to more than 1 billion at-risk people each year can empower women, develop infrastructure and combat discrimination against disability. Interventions to curb mosquito-borne NTDs contribute to the goals of urban sustainability and resilience to climate change, while the safe use of insecticides supports the goal of sustainable ecosystems. Although indirectly, interventions to control water- and animal-related NTDs can facilitate the goals of small-scale fishing and sustainable hydroelectricity and biofuels. It is important to note, however, that not all NTD programs will, or can, engage on all SDGs at all levels. Some of the examples cited will occur in only a small number of communities in a selection of countries.

**Table 1:** The sustainable development goals, the linkage between SDGs and NTDs and the NTDs referenced

SDG	NTD-SDG linkage	NTDs referenced
1 - End poverty in all its forms everywhere.	NTDs and the medical poverty trap: Debilitating diseases cause significant economic burden to affected individuals, households and communities. Free of charge and community directed interventions in addition to preventive care can reduce this burden.	Dengue, Chikungunya, Lymphatic filariasis, onchocerciasis Leishmaniasis, Buruli Ulcer, Cysticercosis. Trachoma, Schistosomiasis
2 - End hunger, achieve food security and improved nutrition and promote sustainable agriculture.	Worm infections reduces nutrient uptake in human and animal hosts while infections from other NTDs reduces ability to work. Treating the affected can improve food security and ensure healthy livestock.	STH, schistosomiasis, Chagas, Dracunaliasis, trachoma, lymphatic filariasis, onchocerciasis, HAT, dracunculiasis
3 - Ensure healthy lives and promote well-being for all	NTDs affect all aspects of healthy lives. The promotion of Universal Health Coverage as part of NTD interventions ensures well-being beyond the NTDs.	All NTDs
4 - Ensure inclusive and equitable quality education and promote lifelong learning opportunities for all.	NTDs can cause stigma and reduce school attendance, performance and cognitive ability. School based programs, including deworming and health education, ensure children can attend school.	Lymphatic filariasis, cutaneous leishmaniasis, STH, Schistosomiasis,
5 - Achieve gender equality and empower all women and girls	NTDs disproportionally impact the health of girls and women. Community directed distribution programs and women’s groups as part of drug distribution programs can empower women.	STH, Zika, Chagas, Schistosomiasis, onchocerciasis, lymphatic filariasis
6 - Ensure access to water and sanitation for all.	Water and sanitation play an important role in the lifecycle of most NTDs. The joint WASH-NTD strategy aims to improve access to water and sanitation for the most disadvantaged populations.	STH, Dracunculiasis, Trachoma, Schistosomiasis, Onchocerciasis, Lymphatic filariasis, Dengue, Chikungunya
7 - Ensure access to affordable, reliable, sustainable and modern energy for all	Construction of dams can serve as breeding sites while biogas material used can be infectious. Impact of dams on water tables can change vector ecology. Vector control and the disinfection of biogas material can leverage the proliferation of rural sustainable energy projects.	Schistosomiasis, Onchocerciasis, Lymphatic filariasis
8 - Promote inclusive and sustainable economic growth, employment and decent work for all	NTD infections are a large burden to health care system and reduce economic productivity of the workforce. Deworming and sustained vector control ensures limited impact on health care system and a healthy workforce.	STH, lymphatic filiarisis, rabies, dengue, hookworm, schistosomiasis,
9 - Build resilient infrastructure, promote sustainable industrialization and foster innovation	Interventions to the most neglected populations requires development and investment in supply chains including transport and storage infrastructure as well as clinics for distribution of donated medicines.	STH, Dracunculiasis, Trachoma, onchocerciasis, Schistosomiasis, Lymphatic filariasis
10 - Reduce inequality within and among countries.	Inequalities in disease prevalence across socioeconomic groups is a hallmark of NTDs. Interventions aimed at the most disadvantaged and marginalized populations aims to reduce this inequality.	All NTDs
11 - Make cities inclusive, safe, resilient and sustainable	Mosquito and other disease vectors have adapted and proliferated in urban environments. Actively removing breeding sites through community based interventions ensures cities are resilient to this threat.	Leishmaniasis, Chagas, Dengue, Chikungunya, Zika, Yellow Fever
12- Ensure sustainable consumption and production patterns	Chemicals are frequently used to combat nuisance or disease vector mosquitos. The sustainable use and management of chemicals is ensured through continued pesticide safety and efficacy evaluation.	Dengue, Chikungunya, Zika, Yellow Fever
13 - Take urgent action to combat climate change and its impacts	Vector borne disease epidemics increase with changes in temperature, rainfall and relative humidity. A better understanding of the effect of climate change can be gained through modelling and surveillance of the interaction between NTDs and climate.	Dengue, Chikungunya, Zika, Yellow Fever, schistosomiasis, lymphatic filariasis
14 - Conserve and sustainably use the oceans, seas and marine resources	Clean water bodies are important to maintain food security and good sanitation. NTD interventions can address contaminated water through treatment programs and by providing educational service to affected communities.	Trematodes
15 - Sustainably manage forests, combat desertification, halt and reverse land degradation, halt biodiversity loss	Deforestation leads to the proliferation of vector borne disease affecting people who work or live at interface with forest. Active vector control and providing educational service to affected communities can mitigate the impact of disease vector proliferation.	Dengue, Chikungunya, Zika, Yellow Fever, cutaneous leishmaniasis, loiasis,
16 - Promote just, peaceful and inclusive societies	NTD disease epidemics frequently occur during times of war and crisis. Advocating for interventions to affected populations in times of crisis can be used as a tool to promote peace.	Dracunculiasis, leishmaniasis
17 - Revitalize the global partnership for sustainable development	Public–private partnerships for NTD interventions have been key and effective. Experience gained from working with these partnerships can be used to build on partnerships for other SDG themes.	All NTDs

To measure the progress of the NTD target in the context of the SDG goal, the WHO will be tracking and reporting the number of people requiring interventions against neglected tropical diseases. In addition, the WHO will monitor NTDs in the context of a few targeted SDGs. Most notably, within the health target, the WHO will use NTD indicators to monitor equity towards the goal of Universal Health Coverage. Beyond the health goal, but critical to the NTD agenda, NTDs will be used to track progress on the “Provision of Clean Water”, “No Poverty”, “Zero Hunger”, “Quality Education” and “Sustainable Cities and Communities” targets.

Remarkable progress has already been made towards achieving the end of the epidemic of NTDs even without the financial support seen for other disease interventions. Importantly, the strong political will to address the suffering of the most marginalized populations has meant that programs were able to have an impact even during financial crisis and conflicts. The challenges presented by NTDs require the multi-sectoral responses encouraged by the 2030 Agenda. This should remind the NTD community to think differently about the impact their interventions have, and be proactive in working across sectors and disciplines to ensure progress towards sustainable development.

## Additional file

Additional file 1: Multilingual abstracts in the five official working languages of the United Nations. (PDF 474 kb)

## Abbreviations

MDA: Mass drug administration
MDGs: Millennium development goals
NTDs: Neglected tropical diseases
SDGs: Sustainable development goals
STH: Soil-transmitted helminths
WASH: Water, sanitation and hygiene
