# Supplementary material for: The cross-cutting contribution of the end of neglected tropical diseases to the sustainable development goals
Source: Infect Dis Poverty. 2017 Apr 4;6:73. doi: 10.1186/s40249-017-0288-0 (PMC5379574; doi:10.1186/s40249-017-0288-0)
Supplement: Additional file 1: — Multilingual abstracts in the five official working languages of the United Nations. (PDF 474 kb) [file 40249_2017_288_MOESM1_ESM.pdf]

Translation of the abstract into the five official working languages of the United Nations

المساهمة الشاملة لنهاية أمراض المناطق المدارية المنسية في تحقيق أهداف التنمية المستدامة

ماتيو بانجرت، ديفيد اتش مولينو، ستيف ووكر ليندساي، كريستوفر فيتزباتريك وديرك إنجلز

الملخص
أهداف التنمية المستدامة (SDGs) تدعو إلى استجابة متكاملة، من النوع الذي قاد جهود أمراض المناطق المدارية المهملة في العقد الماضي.
التدخلات في شأن الأمراض المدارية المهملة لها أعظم أهمية لـSDG3: الهدف الصحي، حيث التركيز على الحقوق، والالتزام بالوصول إلى من هم في حاجة للخدمات الصحية، أينما كانوا يقيمون ومهما كانت ظروفهم، تتماشى بشكل أساسي مع هدف التغطية الصحية الشاملة. ومع ذلك، التدخلات في شأن الأمراض المدارية المهملة، تؤثر أيضا على وتتأثر بالعديد من المجالات التنموية الأخرى التي يشملها جدول أعمال عام 2030. الاستراتيجيات مثل العلاج الدوائي الشامل أو الإدماج البرنامجي لأنشطة الأمراض المدارية المهملة، والمياه والصرف الصحي والنظافة (SDG6) تدفعها شراكات عالمية فعّالة (SDG17). بإمكان التدخلات بشأن الأمراض المدارية المهملة أن يكون لها أثر على الفقر (SDG1) والجوع (SDG2)، كما يمكنها تحسين التعليم (SDG4) والعمل والنمو الاقتصادي (SDG8)، وبالتالي الحد من عدم المساواة (SDG10). عملية توزيع الأدوية المتبرع بها بقيادة مجتمعية إلى أكثر من مليار شخص *يعزز* تمكين المرأة (SDG5)، والبنية التحتية اللوجستية (SDG9) وعدم التمييز ضد الإعاقة (SDG16).
التدخلات للحد من الأمراض المدارية المهملة التي تنتقل عن طريق البعوض تساهم في تحقيق أهداف الاستدامة الحضرية (SDG11) والقدرة على التكيف مع تغير المناخ (SDG13)، في حين أن الاستخدام الآمن للمبيدات يدعم هدف النظم الإيكولوجية المستدامة (SDG15). التدخلات للسيطرة على الأمراض المدارية المهملة المنقولة بالمياه والمتعلقة بالحيوانات يمكنها، عن طريق غير مباشر، أن تسهل أهداف الصيد على نطاق صغير (SDG14) والطاقة الكهرومائية والوقود الحيوية المستدامة (SDG7).
الأمراض المدارية المهملة تتكاثر في المناطق الأقل نموا في البلدان من جميع درجات الدخل، المناطق حيث أعداد كبيرة من الناس يحصلون على القليل أو لا يحصلون على الرعاية الصحية المناسبة، أو المياه النظيفة والصرف الصحي والإسكان والتعليم والنقل والمعلومات. ويقيم هذا الاستعراض الاستطلاعي كيف يمكن لإنهاء وباء الأمراض المدارية المهملة في هذا السياق أن تؤثر على ويحسن الفرص لتحقيق أهداف التنمية المستدامة.

Translated from English version into Arabic by SAlkhodair, through

TRANSLATORS
WITHOUT BORDERS

被忽视的热带病对可持续发展目标的跨领域贡献

Mathieu Bangert, David H. Molyneux, Steve W. Lindsay, Christopher Fitzpatrick and Dirk Engels

摘要
十年来，致力于被忽视热带病（NTD）的行动表明，可持续发展目标（SDGs）也需要采取综合应对措施。
NTD 干预措施与 SDG3−健康目标有很大的相关性，SDG3 部分指标关注公平，承诺人们得到卫生服务，无论他们在哪里生活，无论其情况如何，都是“全民健康覆盖”目标的对象。然而，NTD 干预措施也影响到了 2030 议程所涵盖的许多其他发展领域并受其影响。诸如全民服药或 NTD 和 WASH 行动（SDG6）的计划整合等战略是由有效的全球伙伴关系（SDG17）来推动的。NTD 干预措施也可影响贫困（SDG1）和饥饿（SDG2），优化教育（SDG4），

繁荣工作和经济（SDG8），从而减少不平等（SDG10）。10 亿受赠药品的群众的社区主导分布情况显示了妇女赋权（SDG5），物流基础设施（SDG9）和公平对待残疾人（SDG16）。干预蚊传 NTD 干预有助于实现城市可持续性发展（SDG11）和提高应对气候变化的能力（SDG13），而安全使用杀虫剂则支持可持续生态系统的目标（SDG15）。控制水源和动物相关 NTD 的干预措施可以促进实现减小捕捞规模（SDG14）以及可持续水电和生物燃料（SDG7）的目标。
NTD 在经济欠地区（按收入划分）扩散，大量人口缺乏足够的保健、清洁水、卫生设施、住房、教育、运输和信息。本堪域综述评估了在这种情况下终止 NTD 传播如何影响和实现可持续发展目标。

Translated from English version into Chinese by Men-Bao Qian, through

CHINESE CENTER FOR DISEASE CONTROL AND PREVENTION
NATIONAL INSTITUTE OF PARASITIC DISEASES

La contribution transversale de la fin des Maladies Tropicales Négligées aux Objectifs de Développement Durable

Mathieu Bangert, David H. Molyneux, Steve W. Lindsay, Christopher Fitzpatrick et Dirk Engels

Résumé
Les Objectifs de développement durable (ODD) exigent une réponse intégrée, tout comme celle ayant définie les efforts à consentir pour éradiquer les Maladies Tropicales Négligées (MTNs) au cours de la dernière décennie.
Les interventions autour des MTNs sont particulièrement pertinents pour l' ODD-3, l'objectif de santé, pour lequel l'accent est mis sur l'équité, et dont l'engagement à atteindre les personnes nécessitant des services de santé, où qu'elles vivent et quelle que soit leur situation, est fondamentalement aligné avec l'objectif d'une Couverture Maladie Universelle . Néanmoins, les interventions liées aux MTNs affectent et sont affectées par de nombreux autres domaines du développement couverts par l'Agenda 2030. <Des stratégies telles que l'administration massive de médicaments ou l'intégration programmatique des MTNs et des activités WASH (eau, assainissement et hygiène) (ODD-6) sont menées par des partenariats efficaces au niveau mondiaux (ODD-17). L'intervention contre les MTNs peut également avoir un impact sur la pauvreté (ODD-1), la sous-alimentation (ODD-2), mais également améliorer l'éducation (ODD-4), le travail et la croissance économique (ODD-8), réduisant ainsi les inégalités (ODD-10). La distribution menée par la communauté de dons de médicaments à plus de un milliard de personnes renforce l'émancipation des femmes (ODD-5), les infrastructures logistiques (ODD-9) et la non-discrimination à l'égard des personnes handicapées (ODD-16). Les interventions visant à freiner les MTNs transmises par les moustiques contribuent aux objectifs de développement urbain durable (ODD-11) et de résilience au changement climatique (ODD-13), tandis qu'une utilisation raisonnée des insecticides appuie l'objectif d'écosystèmes durables (ODD-15). De façon plus indirecte, les interventions visant en endiguer les MTNs transmises par l'eau et les animaux peuvent faciliter l'atteinte des objectifs de pêche à petite échelle (ODD-14), de production d'hydroélectricité et de biocarburants durables (ODD-7).

Les MTNs prolifèrent dans les zones moins développées de nombreux pays - quelle que soit son échelle de revenus. Il s'agit de zones où un grand nombre de personnes ont peu, voire aucun accès à des services de santé adéquats, à l'eau potable, l'assainissement, le logement, l'éducation, les transports et l'information. Cette étude évalue comment mettre un terme à l'épidémie des maladies tropicales négligées dans ce contexte pourrait avoir un impact et améliorer les perspectives d'atteinte des ODDs.

Translated from English version into French by Jean Bellefleur, through

TRANSLATORS
WITHOUT BORDERS

Многоплановая роль ликвидации «забытых» тропических болезней в достижении целей устойчивого развития

Матье Бангерт, Дэвид Х. Молинье, Стив В. Линдсей, Кристофер Фицпатрик и Дирк Энгельс (Mathieu Bangert, David H. Molyneux, Steve W. Lindsay, Christopher Fitzpatrick and Dirk Engels)

Аннотация

Достижение целей устойчивого развития (ЦУР) требует комплексного подхода. Именно такой подход определил направление предпринимавшихся на протяжении последних десяти лет усилий по борьбе с «забытыми» тропическими болезнями (ЗТБ).

В наибольшей степени меры, направленные на ликвидацию ЗТБ, содействуют достижению цели в области здравоохранения (ЦУР 3), в которой пристальное внимание уделяется вопросам обеспечения равенства и обязательствам по предоставлению медицинской помощи всем в ней нуждающимся, где бы они ни жили и в каких бы обстоятельствах ни находились, что в полной мере соответствуют задаче обеспечения всеобщего охвата медицинской помощи. При этом меры по борьбе с ЗТБ оказывают влияние и на многие другие области, составляющие предмет Повестки дня на период до 2030 года, равно как и сами подвергаются воздействию. Применяемые стратегии, в частности, организация массированного применения лекарственных препаратов и интеграция программ по борьбе с ЗТБ и программ, реализуемых в рамках инициативы «Водоснабжение, санитария и гигиена для всех» (ЦУР 6), опираются на эффективные партнерские связи, сформированные на глобальном уровне (ЦУР 17). Кроме того, меры по борьбе с ЗТБ могут повлиять на сокращение масштабов бедности (ЦУР 1) и голода (ЦУР 2), способствовать усовершенствованию образования (ЦУР 4), обеспечению занятости и экономическому росту (ЦУР 8) и тем самым сократить масштабы неравенства (ЦУР 10). Регулируемое на уровне общины распределение пожертвованных лекарственных средств позволило охватить более 1 миллиарда людей, что способствует расширению прав и возможностей женщин (ЦУР 5), укреплению логистической инфраструктуры (ЦУР 9), недопущению дискриминации в отношении людей с ограниченными возможностями (ЦУР 16). Меры, нацеленные на ограничение распространения ЗТБ, переносчиками которых являются комары, способствуют

достижению целей, связанных с устойчивыми городами (ЦУР 11) и устойчивостью к изменениям климата (ЦУР 13), в то время как безопасное применение инсектицидов способствует достижению цели, связанной с устойчивыми экосистемами (ЦУР 15). Меры по борьбе с ЗТБ, источником которых являются вода и животные, могут, пусть и опосредованно, способствовать достижению целей, связанных с маломасштабным рыболовством (ЦУР 14) и устойчивостью в гидроэнергетике и биотопливной отрасли (ЦУР 7).
ЗТБ распространены в наименее развитых районах стран с различными уровнями доходов. Доступ значительного количества жителей таких районов к адекватным услугам здравоохранения, чистой воде, санитарным системам, жилью, образованию, транспорту и информации ограничен либо отсутствует. Настоящий обзорный анализ позволяет оценить, каким образом в сложившихся условиях ликвидация эпидемии ЗТБ может повлиять на достижение ЦУР и улучшить ситуацию в этом направлении.

Translated from English version into Russian by Alexander Poddubnyy, through

TRANSLATORS
WITHOUT BORDERS

La contribución intersectorial de la erradicación de las Enfermedades Tropicales Desatendidas a los Objetivos de Desarrollo Sostenible

Mathieu Bangert, David H. Molyneux, Steve W. Lindsay, Christopher Fitzpatrick y Dirk Engels

Resumen
Los Objetivos de Desarrollo Sostenible (ODS) requieren de una respuesta integrada, del tipo que ha definido los esfuerzos contra las Enfermedades Tropicales Desatendidas (ETD) en la última década. Las intervenciones contra las ETD tienen la mayor relevancia para el ODS3, el objetivo de la salud, donde el enfoque de equidad y su compromiso de llegar a las personas que necesitan servicios de salud, dondequiera que vivan y sean cuales sean sus circunstancias, está fundamentalmente alineado con la meta de cobertura universal de salud . Las intervenciones contra las ETD, sin embargo, también afectan y son afectadas por muchas de las otras áreas de desarrollo cubiertas por la Agenda 2030. Estrategias como la administración masiva de medicamentos o la integración programática de las ETD y de actividades en relación con el agua, el saneamiento y la higiene (ODS6) son impulsadas por asociaciones mundiales eficaces (ODS17). La intervención contra las ETD también puede tener un impacto en la pobreza (ODS1) y el hambre (ODS2), puede mejorar la educación (ODS4), el trabajo y el crecimiento económico (0DS8), reduciendo así las desigualdades (ODS10). La distribución liderada por la comunidad de medicamentos donados a más de mil millones de personas refuerza el empoderamiento de las mujeres (ODS5), la infraestructura logística (ODS9) y la no discriminación contra la discapacidad (ODS16). Las intervenciones para frenar las ETD transmitidas por mosquitos contribuyen a los objetivos de la sostenibilidad urbana (ODS11) y la resistencia al cambio climático (ODS13), mientras que el uso seguro de insecticidas apoya el objetivo de ecosistemas sostenibles (ODS15). Aunque de forma indirecta, las intervenciones para

el control de las ETD relacionadas con al agua y los animales pueden facilitar los objetivos de la pesca artesanal (ODS14) y la hidroelectricidad y biocombustibles sostenibles (ODS7).
Las ETD proliferan en las zonas menos desarrolladas de los países en toda la gama de los ingresos, áreas en las que un gran número de personas tienen poco o ningún acceso a un servicio de salud adecuado, agua potable, saneamiento, vivienda, educación, transporte e información. Esta revisión de alcance evalúa como, en este contexto, poner fin a la epidemia de las ETD puede repercutir y mejorar nuestras posibilidades de alcanzar los ODS.

Translated from English version into Spanish by Ana Elvia Carrasco-Bustillos, through

TRANSLATORS
WITHOUT BORDERS
